# Antioxidants in Longevity and Medicine

**DOI:** 10.1155/2013/820679

**Published:** 2013-11-12

**Authors:** Nilanjana Maulik, David McFadden, Hajime Otani, Mahesh Thirunavukkarasu, Narasimham L. Parinandi

**Affiliations:** ^1^Department of Surgery, University of Connecticut Health Center, Farmington, CT-06030, USA; ^2^Department of Internal Medicine, Kansai Medical University, Moriguchi City 570-8507, Japan; ^3^Division of Pulmonary, Allergy, Critical Care, and Sleep Medicine, Dorothy M. Davis Heart & Lung Research Institute, The Ohio State University College of Medicine, Columbus, OH 43210, USA

Oxidative stress has emerged as a critical player in the pathogenesis and pathophysiology of many diseases in experimental animal models and humans. The oxidative stress theory of aging emphasizes that a progressive and irreversible escalation of oxidative damage caused by reactive oxygen species (ROSs) exerts severe influence on the critical aspects of the biology of aging and contributes to impaired physiological functions, increased incidence of diseases, and shortening of life span. The focus of this special issue is to identify avenues that affect both the life span and age-related diseases in humans. In order to accomplish this, experts in the field have contributed original research articles and reviews focusing on the current state of understanding of the molecular pathology of oxidative stress underlying the biology of aging and age-related diseases and development of strategies to alleviate or treat these conditions.

S. Gazman-Beltran et al. demonstrate that a natural antioxidant, nordihydroguaiaretic acid (NDGA) attenuates the oxidative stress-induced decrease of CD33 expression in human monocytes, which is decreased in diabetic patients under oxidative stress. The antioxidant mechanism of NDGA appears to be multifactorial and includes the increased availability of glutathione and activation of nuclear factor E2-related factor-2 (Nrf2), which is known to orchestrate antioxidative and cytoprotective responses to oxidative stress. NDGA appears to be an effective antioxidant to prevent chronic inflammation induced by monocytes.

Recently, studies conducted with dietary polyphenols on neurological disorders have shown promising results. K. S. Bhullar and H. P. V. Rupasinghe have reviewed the effect of polyphenols on age-related neurological disorders and discussed the role of polyphenols in multiple signaling pathways, including PI3K/Akt, Nrf2/HO1 PPAR, STAT, NF-*κ*B, HIF, and MAPK. All of the pathways can alleviate oxidative damage and inflammation. This review article offers insights into the complex interactions of polyphenols with intracellular signaling pathways in neuronal cells and provides novel approach to neurological disorders.

Resveratrol is a calorie restriction (CR) mimetic agent. CR reverses many of the obesity-related increases in inflammatory cytokines. I. Zagotta et al. have reported that PAI-1 gene expression is increased in adipose tissue in obese subjects, and this is critically involved in chronic inflammation of blood vessels that leads to atherosclerosis and cardiovascular disease. PAI-1 gene is found to be upregulated due to inflammatory conditions associated with obesity. Resveratrol treatment has been shown to suppress the PAI-1 gene in human inflamed adipose tissue. Interestingly, the effect of resveratrol on PAI-1 is not mediated by modulation of PI3 K/Akt, SIRT1, AMPK, and Nrf2 or reactive oxygen species (ROS) but proceeds through the inhibition of the NF-*κ*B pathway. This suggests that resveratrol inhibits the generation of inflammatory cytokines in the adipose tissue via a novel anti-inflammatory pathway.

Advanced glycation end products (AGEs) play a crucial role in senescence. B. Buttari et al., have demonstrated that resveratrol may be effective in treating autoimmune diseases that are intimately related to the activation of dendritic cells (DCs) with the AGEs. B. Buttari et al. have also shown that resveratrol pretreatment of human monocyte-derived DCs prevents the activation of DCs in response to the AGE-albumin. The mechanism is by inhibiting the DC maturation and proinflammatory cytokine expression associated with the inhibition of NF-*κ*B activation. Because the specific receptor for AGE (RAGE) is down-regulated in the resveratrol-pretreated DCs, resveratrol inhibits oxidative stress and inflammatory response at the level of RAGE upstream of PI3K/Akt, MAPK, or NF-*κ*B. This finding is interesting since the NF-*κ*B pathway is modulated by resveratrol in other biological systems. This unique pharmacological action of resveratrol on DCs that are activated by AGEs is a subject for further investigation.

ROS derived from the mitochondria activates the redox-sensitive transcriptional factor, nuclear respiration factor-2 (NRF2). NRF2-induced mitochondrial biogenesis improves the function of electoral transfer chain (ETC) and prevents ROS generation in mitochondria. Thus, the agents that can activate NRF2 are attractive in ameliorating oxidative stress-induced pathology. X. Miao et al. have demonstrated that an NRF2 activator MG132 has prevented the aortic injury in type-1 diabetic mice, suggesting that activation of NRF2 represents a promising approach for hyperglycemia-induced vascular complications.

Aging is related to circadian rhythm disruption. The concept of chrononutrition raised by M. Garrido et al. has prompted development of a strategy that takes timing of tryptophan-enriched food intake into account when delivering antioxidative medicine. Chrononutrition studies suggest that along with the content of food, the time of ingestion is essential for the natural functioning of the circadian system, which stimulates melatonin secretion at night. Melatonin produces circadian rhythm and acts as a potent antioxidant and ROS scavenger. It also inhibits ROS generation by preventing the electron leakage from mitochondria, thereby stimulating the antioxidative defense system. Thus, it is anticipated that administration of melatonin either directly or indirectly from food enhances one's antioxidant status.

A review article described by H. Otani opens a new avenue for antioxidative medicine. Although ROS is involved in many pathological phenomena in humans with advanced age, antioxidative medicine is not always successful in alleviating human disease. The limitation of general antioxidants in preventing oxidative stress-induced diseases is attributed to ROS playing a pivotal role in generating redox signaling. This is necessary for maintaining homeostasis and generating adaptive response to lethal oxidative damage. Thus, antioxidants must be more site specific to eliminate only harmful ROS and leave beneficial ROS. Recent new drugs used for treating lifestyle-related diseases such as hypertension, hyperlipidemia, and hyperuricemia possess a pleiotropic effect that is designated as the site-specific antioxidant against endothelial NADPH oxidase and xanthine oxidase. Antioxidants that specifically reduce mitochondrial ROS generation will also be putative drugs to ameliorate age-associated disease and prolong lifespan.

Mitochondrial generation of ROS is intimately related to aging. Eliminating the harmful production of ROS from mitochondria represents a powerful strategy to prevent cellular senescence. The review article authored by K. Shinmura highlights the importance of preventing mitochondrial generation of ROS. Age-related mitochondrial dysfunction is due to oxidative stress-mediated accumulation of somation mutation in mitochondrial DNA. A growing body of evidence suggests that caloric restriction (CR) mediates attenuation of mitochondrial oxidative damage via decreasing the production of mitochondrial ROS rather than by enhancing the antioxidant defense. K. Shinmura suggests that sirtuin might be involved in the CR-induced attenuation of mitochondrial oxidative damage. This review article concludes that CR-mimetic agents, such as resveratrol and sirtuin activators, are promising agents to improve the mitochondrial function and prolong lifespan, although their exact mechanism(s) of action should be carefully examined.

Myocardial reperfusion injury is mediated at least in part by oxidative stress. Yet, there have been no antioxidants that unequivocally confer benefit in the postischemic heart in the clinical setting, although numerous agents have been shown to exert cardioprotective effects on animal models of ischemia/reperfusion injury. Angelos et al. have demonstrated that the neutrophil esterase inhibitor sivelestat reduces the infarct size and improves cardiac function in an isolated rat heart that lacks neutrophils. The cardioprotective effect of sivelestat appears to be mediated by nitric oxide, which has consistently been implicated in the cardioprotection against myocardial ischemia/reperfusion injury. Future clinical trials using sivelestat are warranted to test whether this clinically available drug is indeed effective in ameliorating myocardial reperfusion injury in humans.

G. Grosso et al. have advocated in favor of the health benefits of red orange on the basis of research on experimental models and epidemiological evidence. The authors argue that the beneficial effects of the red orange fruit may be exerted through the synergistic actions of the natural products present in the fruit. These natural products possess antioxidant actions, which may protect against oxidative damage, and the total beneficial actions of all the natural products (antioxidants) present in the red orange fruit could be more effective than an individual antioxidant.

The lack of mitochondrial superoxide dismutase (Mn-SOD) leading to the loss of hearing during aging has been clearly demonstrated by M. Kinoshita et al. With the use of Mn-SOD heterozygous knockout mice as the experimental model of mitochondria-derived oxidative stress, the authors have shown that loss of Mn-SOD by half appears to elevate the oxidative stress in the cochlea to a certain degree, but that may not be adequate to speed up the age-induced damage of the cochlea. Nevertheless, the role of Mn-SOD in the mitochondria that scavenges the superoxide radical in the oxidative stress that leads to loss of hearing during aging has been attempted in this study.

Z. Makpol et al. have studied the gene expression-modulating actions of gamma-tocotrienol in the senescent human diploid fibroblasts by utilizing the microarray analysis. This study has emphasized on the lipid-soluble antioxidant (gamma-tocotrienol) actions on the modulation of genes associated with the cellular aging and oxidative stress. This study has demonstrated that gamma-tocotrienol appears to block cellular aging of human fibroblasts through the modulation of gene expression in the cells. In addition, the role of lipid peroxidation in cellular aging and associated gene expression is apparent from this study.

D. McCormack and D. McFadden have discussed in depth the antioxidant and disease-modifying actions of pterostilbene. The antioxidant actions of pterostilbene have been connected with cancer protection, treatment of neurological diseases, suppression/treatment of inflammation, attenuation of vascular diseases, and improvement of diabetes. The authors have provided an excellent account on the clinical use of pterostilbene in prevention/treatment of several disorders and diseases.

A. B. Rodriguez et al. showed that fish oils containing the omega-3 polyunsaturated fatty acids (n3-PUFA) are capable of attenuating the levels of inflammatory cytokines and oxidative stress markers in the serum of multiple sclerosis patients. From this study, the authors have concluded that the fish oil supplementation to the multiple sclerosis patients effectively lowers the levels of inflammatory cytokines and nitric oxide (oxidant), thus leading to the protection against multiple sclerosis.

J. Rosado-Perez et al. have shown that physical exercise like Tai Chi offers beneficial effects in lowering oxidative stress in humans. In their study on Mexican adults, the authors have revealed that the subjects who have practiced Tai Chi have exhibited lower extent of lipid peroxidation and elevated superoxide dismutase activity as compared to the control individuals. From this study, the authors have concluded that Tai Chi is more effective in elevating the antioxidant status in humans as compared to walking.

S. Dande et al. have discussed on the natural antioxidant actions of the commonly consumed foods of plant origin in India. In this review, the authors have emphasized on the domestic processing of plant foods that could affect the quality of the plant foods that are consumed. The authors discussed the importance of polyphenols present in the plant foods that could act as natural antioxidants. The authors have stated that the domestic processing of plant foods may not alter the content of polyphenols and antioxidant activities in the plant foods.

In a study on the hypoxia-induced neuroinflammation, Z. Wu et al. have shown that the Brazilian green propolis inhibits the NF-*κ*B activation in the microglia. The authors have demonstrated that the mitochondrial ROS formation is crucial for the activation of NF-*κ*B activation in the microglia. Therefore, it appears that propolis could have also acted as an antioxidant in attenuating the hypoxia-induced neuroinflammation in the microglia.

A. L. Zagayko et al. have shown that the grape polyphenols increase the activity of high-density lipoprotein (HDL) enzymes in old and obese rats. The authors have revealed that the activity of paraoxonase, lecithin: cholesterol acyltransferase (LCAT) in plasma have been lowered and the cholesterol ester transfer protein (CETP) activity has been elevated in old rats. This study emphasizes the actions of grape polyphenols on the elevation of HDL enzymes during aging.

These papers represent an exciting and insightful snapshot of current antioxidant research. State-of-the-art, exciting challenges, which still present many new challenges, are highlighted in this special issue, which may inspire and help advance antioxidant research. We would like to thank all authors, reviewers, and the editors for producing this special issue.

Antioxidative medicine has become a practical tool to treat age-associated disease and to prolong the lifespan since oxidative stress is critically involved in human aging. The purpose of the topics covered in this special issue is to provide additional opportunities to all the investigators in the field in order to identify avenues that impact both the life span and age-related diseases in humans. Therefore, the contributions made by the experts in this issue as original research articles and reviews will hopefully stimulate the continuing efforts of the investigators to understand and establish the molecular pathology of oxidative stress underlying the biology of aging and age-related diseases and development of strategies to alleviate or treat these conditions.

*Nilanjana Maulik* *Nilanjana Maulik* *David McFadden* *David McFadden* *Hajime Otani* *Hajime Otani* *Mahesh Thirunavukkarasu* *Mahesh Thirunavukkarasu* *Narasimaham L. Parinandi* *Narasimaham L. Parinandi*

